# Open TSST VR: Psychobiological reactions to an open version of the Trier Social Stress Test in virtual reality

**DOI:** 10.3758/s13428-025-02662-x

**Published:** 2025-04-15

**Authors:** Katrin Linnig, Saskia Seel, Bernadette von Dawans, William Standard, Daniel Zielasko, Benjamin Weyers, Gregor Domes

**Affiliations:** 1 Biological and Clinical Psychology, Department of Psychology, University of Trier, Johanniterufer 15, 54290 Trier, Germany; 2 Institute for Cognitive and Affective Neuroscience, University of Trier, Trier, Germany; 3 Computer Science, Human–Computer Interaction, University of Trier, Trier, Germany

**Keywords:** Trier social stress test, Virtual reality, VR, Hypothalamic–pituitary–adrenal axis, HPA axis, Cortisol, Alpha amylase

## Abstract

The Trier Social Stress Test (TSST) has become one of the most frequently employed laboratory stressors in human studies over the past decades. Several TSST adaptations for the presentation in virtual reality (VR) have been introduced and evaluated recently. Here, we describe a freely available version, the Open TSST-VR. In two independent studies, we evaluated subjective, endocrine, and heart rate responses to the Open TSST-VR compared to an active control condition. In study 1, 50 men were randomly assigned to the Open TSST-VR or the control condition. Compared to the active control condition, the Open TSST-VR induced higher levels of subjective stress and significantly more cortisol responders. In study 2, we employed a balanced within-subject design comparing groups of 46 men and women. Again, the TSST-VR induced more stress than the control condition and a stronger cortisol response, but there were also order effects suggesting that the TSST-VR is less suitable for within-subject comparisons. In both studies, we observed a substantial stress level (and cortisol responders) in the control condition indicating that future studies should further elaborate on non-stressful control conditions, either without any task or non-stressful active components. Overall, the Open TSST-VR is a versatile tool for collaborative experimental stress research that offers flexibility to a broad range of future research questions among various disciplines.

## Introduction

Acute psychosocial stress triggers several psychophysiological responses, such as the activation of the sympathetic–adrenal–medullary system (SAM system) and the hypothalamic–pituitary–adrenal axis (HPA axis), which promote the individual’s adaptation to a stressful situation. To investigate the effects of acute stress and modulating factors on the HPA axis and other physiological systems, standardized stressors have been applied to induce moderate stress in a laboratory setting. Different stress induction procedures have been developed and evaluated for this purpose. One of the most frequently administered laboratory stressors is the Trier Social Stress Test (TSST; Kirschbaum et al., 1993). The combination of a mock job interview and mental arithmetic task in front of a panel of two or three judges is perceived as uncontrollable and socially threatening by most participants, leading to a robust endocrine stress response (Dickerson & Kemeny, 2004). However, the TSST has its drawbacks: It requires at least two trained judges, several adjacent rooms, and is potentially prone to protocol variations (Goodman et al., 2017).

The increasing affordability of virtual reality (VR) systems and VR’s associated experimental potential and advantages have led to steady growth in employing this technology for psychological treatments (Valmaggia et al., 2016) as well as in psychosocial research. Various paradigms have been developed and used in stress research to induce psychological stress in virtual environments (for a meta-analysis see Dammen et al., 2022), e.g., presentation tasks (Kothgassner et al., 2016; Shiban et al., 2016; Zimmer et al., 2019a), or other challenging environments adapted to VR (Dibbets, 2020; Rodrigues et al., 2021).

VR adaptations of the TSST therefore have the advantage of being less costly and of potentially benefitting the procedure’s standardization and comparability across sessions and labs. Moreover, the TSST-VR enables individual elements to be varied quite easily (e.g., the panel’s characteristics; e.g., Halbeisen et al., 2023) as well as the set of additional variables, e.g., head and eye movements, as long as an eye tracker is available in the HMD (Vatheuer et al., 2021). Therefore, the number of studies using VR adaptations of the TSST has increased since the first publications in 2010 (Jönsson et al., 2010; Ruiz et al., 2010). Most of these studies used their own developed software to implement the TSST in VR, and it remains unclear how these VR versions of the TSST differ in the elicited endocrine stress response (Helminen et al., 2019). These initial studies differ in several aspects, including the hardware used to create the VR environment (head-mounted displays vs. cave automatic virtual environments), the general graphic quality of the presentation, and realism of the avatars’ modeling and animation. We can assume that these aspects contribute to the immersion and feeling of presence (Slater & Wilbur, 1997), two factors that may play an important role in the effectiveness of VR interventions (Helminen et al., 2019; Oh et al., 2018; Slater et al., 2022). While immersion is achieved by the technology used to create a virtual environment, presence is defined as the subjective feeling of actually being in the virtual environment (Slater & Wilbur, 1997). The potentially high variance of the TSSTs used in VR with regard to immersion and presence impairs these studies’ general comparability and ultimately complicates a comparative interpretation of the results (Dammen et al., 2022).

To promote collaborative research using VR environments for inducing psychosocial stress in the laboratory, we developed a freely available “open” version of the TSST-VR. This Open TSST-VR closely resembles the standard TSST protocol by Kirschbaum et al. (1993) and the placebo version by Het et al. (2009) and comprises a panel consisting of three mixed-sex virtual avatars controlled by the experimenter. The virtual room as well as the avatars were designed with additional animation, e.g., dynamic shadows, and body movements to enhance the environment’s responsivity and consequently the feeling of presence (Garau et al., 2005). In addition, all elements in the virtual environment can easily be adapted to match the participant sample (e.g., the virtual agents’ language spoken and ethnicity) and to suit the research interest (e.g., additional elements in the virtual room).

Here, we report on two independent studies designed to evaluate this new open version of a TSST in VR. In study 1, we evaluated the Open TSST VR in terms of the subjective and psychobiological responses in comparison to an active control condition. Study 2 was conducted to replicate these results in a within-subject design and to examine potential sex differences.

## Study 1: Psychobiological reactions to the Open TSST VR

### Method

#### Participants

Fifty healthy male participants (age range 18–30 years,* M* = 24; *SD* = 2.7) were enrolled in the study. An all-male sample was chosen for this first evaluation to avoid the effects of hormonal changes of the menstrual cycle and oral contraceptives on the HPA axis (Kirschbaum et al., 1999; Kudielka et al., 2009). Based on the effect of η_p_^2^ = 0.087 of the group (VR stress vs. VR control) x time interaction in our previous study with a similar version of the TSST-VR (Zimmer et al., 2019a), the minimum sample size for a within-between interaction of two groups and eight repeated measurements was estimated in G*power (Faul et al., 2007) at *N* = 44 (*f* = 0.30; α = 0.05; 1–β = 0.95; correlation for repeated measures *r* = 0.30; correction for non-sphericity Greenhouse Geisser ε = 0.44). To compensate for possible dropouts, we aimed for a total sample of* N* = 48.

Participants were recruited by on-campus advertisement. Interested individuals were included in the study if they met the following inclusion criteria: BMI between 19 and 29.9 kg/m^2^, age between 18 and 30 years, no acute or chronic somatic or psychiatric disease, no regular intake of medication, no psychotherapeutic treatment during the last year, nicotine intake equivalent to less than five cigarettes per day, and no regular night shifts.

The study was approved by the ethics committee at the University of Trier (application reference number: 45/2017) and conducted in line with the Declaration of Helsinki. All participants gave informed written consent and were paid 50€ for participating.

#### Open TSST-VR

The VR adaptation of the TSST used in this study resembled all the main features of the TSST’s original protocols (Kirschbaum et al., 1993) and an active control condition, the “placebo TSST” (Het et al., 2009). The VR environment was presented in an HMD (VIVE PRO, HTC Corporation, Taoyuan, Taiwan) with integrated headphones. As in the original TSST, a three-person selection panel for a job interview was created in Unity. In this VR version, the middle “active” panel member (male) is controlled by the experimenter and leads the job interview by asking prerecorded questions or giving feedback. The other two passive members (female and male) behave automatically, e.g., taking notes and looking at the participant from time to time. In the control condition, the virtual experimental room without the panel is presented. For technical details, see the supplementary materials.

Briefly, after calibrating and validating the eye tracker and baseline measures, participants were given instructions for the presentation task: they were either told that they would have to do a job interview in front of a panel of judges who would soon arrive (stress condition; TSST-VR) or that they would have to talk about a self-chosen topic in an empty room (control condition; placebo TSST-VR). In the TSST-VR after a 5-min preparation phase, the active panel member instructed the participant to begin with their presentation, and context-appropriate questions and feedback were then triggered by the experimenter. During the subsequent arithmetic task, the participant had to calculate backwards in steps of 17 starting from 2023 and were interrupted by the active panel member if they made a mistake. In the control condition, participants received the information written on a screen, no feedback was given, and the arithmetic task only consisted in counting upwards in increments of 15 starting from 0. Both periods performing the tasks lasted 5 min in each version. During these procedures in the VR environment, there was no direct interaction between the participants and the experimenter.

#### Questionnaires

**Fig. 1:**
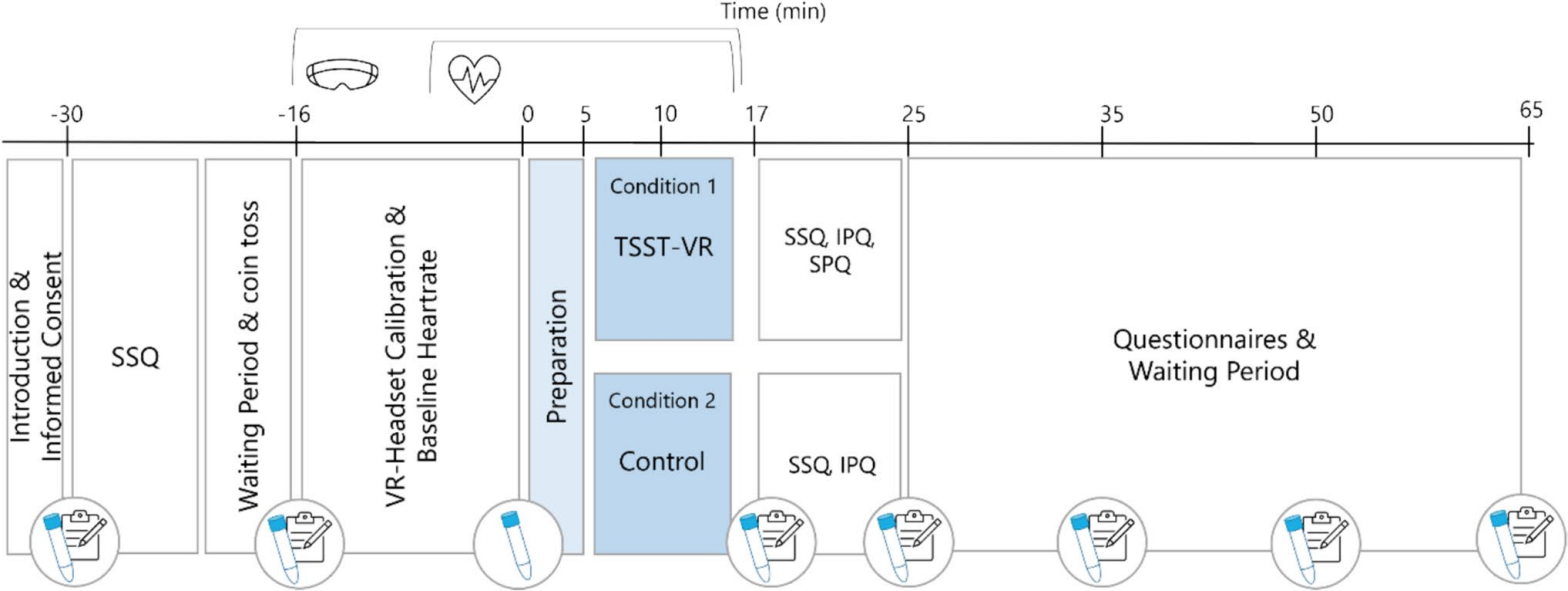
Main procedures of study 1. Timepoint 0 indicates the start of the TSST-VR/Control condition instructions. Heart rate was continuously measured from – 5 to + 16 min. Saliva samples were taken at eight and subjective stress ratings at seven timepoints. *IPQ* Interpersonal Presence Questionnaire, *SPQ* Social Presence Questionnaire, *SSQ* Simulator Sickness Questionnaire

Participants rated their subjective feelings at seven time points on seven visual analog scales (subjective stress, arousal, physical discomfort, perceived control, desire to leave the situation, need for social support, general mood) with a range of 0 (not at all) to 100 (very much) (see Fig. 1) (cf. von Dawans et al., 2012). Additional information about questionnaires used to characterize the study sample is found in the supplementary materials.

#### Saliva sampling and biochemical analysis

We used Salivettes (Sarstedt, Nümbrecht, Germany) to collect saliva at eight time points throughout the experiment (– 30 min, – 16 min, – 1 min, + 17 min, + 25 min, + 35 min, + 50 min, + 65 min, with 0 representing the start of the instructions in VR). Saliva samples were stored at – 18 °C until biochemical analysis. Free salivary cortisol concentrations were determined via time-resolved fluorescence immunoassay (Dressendörfer et al., 1992). To measure the activity of salivary alpha-amylase 2-chloro- 4-nitrophenyl-D maltrotrioside (CNP-G3) was used as the substrate. The enzymatic action of alpha-amylase on this substrate yields 2-chloro-*p*-nitrophenol, which can be spectrophotometrically measured at 405 nm. The amount of alpha-amylase activity present in the sample is directly proportional to the increase in absorbance at 405 nm (Lorentz et al., 1999; Winn-Deen et al., 1988). The intra-assay coefficient of variation (CV) was 4.0–6.7%, the corresponding inter-assay CV was 7.1–9.0% for cortisol and 2.8–6.3% (intra) and 5.5–7.6% (inter) for sAA. Analyses were performed in duplicate and averaged. The saliva sample of one participant at the sampling point at – 16 min was missing and therefore substituted by a sample collected 30 min before the instructions started since the two values showed a close correlation (cortisol: *r* = 0.78, *p* < 0.001; alpha-amylase: *r* = 0.83, *p* < 0.001).

#### Heart rate monitoring

An electrocardiogram was recorded continuously and digitized with a sampling rate of 500 Hz using the ANS Recorder Flex (Neurocor, Fintec GmbH, Gersthofen, Germany). For segmentation of the data stream, markers were set at – 5, 0, + 5, + 11, and + 16 min in reference to the beginning of the TSST preparation phase. Heart rate (HR) was calculated based on RR intervals and averaged over 5-min segments: baseline while standing, preparation phase, and first and second part of the TSST/control condition.

#### Design and procedures

All experimental sessions started at 4:30 p.m. in a randomized between-subject design. Participants were instructed to refrain from physical exercise, alcohol, caffeine, and medications at least 24 h before testing, as well as to refrain from consuming anything but water 2 h prior to testing.

After having given informed consent, the heart rate monitor was attached, and participants were instructed for the saliva collection. They then rated their current symptoms of simulator sickness (SSQ). Then, the experimental condition was determined by a coin toss (– 18 min). Afterward, participants were led into the VR area and put on the HMD. Next, participants sat and stood for the baseline heart rate recording, each lasting 5 min. Once these baseline measurements were taken, the TSST-VR or control condition protocol was started by the experimenter.

After the end of the TSST, the experimenter assisted in taking off the HMD and participants were again asked to rate their current symptoms of simulator sickness (SSQ), their subjective perception of the VR environment, as well as their perception of (social) presence in the TSST (IPQ and SPQ). All participants remained in the laboratory until 65 min after the TSST started and provided another five saliva samples and subjective stress ratings.

#### Statistical analyses

Fisher’s exact tests were used for the statistical comparison of the frequency distributions. Mixed repeated-measure ANOVAs were conducted to test for differences in subjective stress, salivary cortisol, salivary alpha-amylase, and heart rate in both experimental groups (between factors) over the sampling points (within factors). In case of non-sphericity, Greenhouse–Geisser corrected results and ε are reported. Effect sizes for ANOVAs are reported as η^2^_G_. Pairwise comparisons were Bonferroni-corrected. As indicators of a stress response in salivary cortisol, we applied the conservative baseline-to-peak threshold of 2.5 nmol/l (cf. Kirschbaum et al., 1992), with the saliva sample at – 16 min defined as baseline and the individual highest level after the end of the stress induction as peak.

All statistical analyses were done in RStudio version 2023.12.0 with R version 4.3.2 (R Core Team, 2023). For analyses of variance (ANOVA), we used the package *afex* version 1.3–0 (Singmann et al., 2016). All figures were created in GraphPad Prism (version 10.0.0, San Diego, CA). The level of significance was set to *p* < 0.05.

### Results

#### Group differences at baseline, in simulator sickness, presence, and prior experience with VR

There were no group differences regarding self-reported age, BMI, general psychological stress symptoms, fear of negative evaluation, social anxiety, chronic stress, and feeling of personal presence (all *p* > 0.08; see supplementary results Table S1). A mixed ANOVA for symptoms in simulator sickness with *measurement point* as within and *experimental group* as between factor showed that both groups reported more symptoms after than before the VR experience, *F*(1, 48) = 22.18, *p* < 0.001, η^2^_G_ = 0.12, but there were no group differences.

We also examined whether the groups differed in their prior experience with VR. Although there were three persons in the TSST-VR condition who reported frequent use and owned an HMD, using Fisher’s exact tests, we detected no significant differences between the experimental conditions with respect to prior experience (*p* = 0.78) or frequency of the prior VR usage (*p* = 0.28).

#### Subjective reactions

**Fig. 2:**
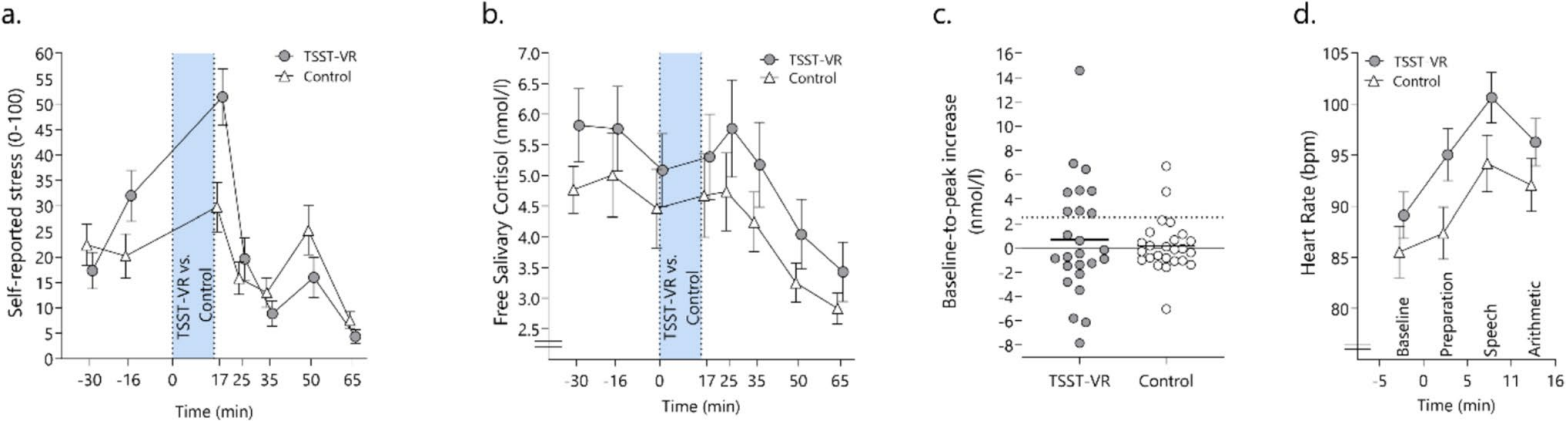
Different facets of the psychobiological stress response to the TSST-VR and the control condition. **a.** Self-reported stress. **b.** Salivary cortisol over the course of the experiment. **c.** Cortisol responder rate (> 2.5 nmol/l). **d.** The heart rate response. *Error bars* represent SEM

For subjective stress level (“How stressed do you feel at the moment”) the mixed ANOVA showed no significant main effect for the experimental *condition, F*(1, 48) = 0.35, *p* = 0.56, η^2^_G_ < 0.01, but a significant main effect *time*, *F*(3.77, 181.19) = 26.63, ε = 0.63, *p* < 0.001, η^2^_G_ = 0.23, and interaction between *condition* and *time, F*(3.77, 181.19) = 6.49, ε = 0.63, *p* < 0.001, η^2^_G_ = 0.07. Pairwise comparisons revealed higher subjective stress + 17 min (after the VR induction) for participants in the TSST-VR compared to the control condition, *t*(48) = – 2.97, *p* < 0.01 (see Fig. 2a), indicating a pronounced stress response in the stress compared to the control condition. All other results on state-related VAS are found in the supplementary materials.

#### Free salivary cortisol

There were more responders (baseline-peak > 2.5 nmol/l) in the TSST-VR (*n* = 9; 36%) compared to the control condition (*n* = 2; 8%), confirmed by a significant Fisher’s exact test (*p* = 0.04). The mixed ANOVA for free salivary cortisol levels revealed a significant main effect for *time*, *F*(2.21, 106.32) = 9.27, ε = 0.32, *p* < 0.001, η^2^_G_ = 0.07, but no main effect for *condition, F*(1, 48) = 1.45, *p* = 0.24, η^2^_G_ = 0.02, or a significant condition*time interaction, *F*(2.21, 106.32) = 0.12, ε = 0.32, *p* = 0.91, η^2^_G_ < 0.001 (Fig. 2b, c).

#### Salivary alpha-amylase

For salivary alpha-amylase, there was also a significant main effect for *time*, *F*(3.88, 186.43) = 4.36, ε = 0.61, *p* < 0.01, η^2^_G_ = 0.02, but neither a main effect for *condition*, *F*(1, 48) = 0.03, *p* = 0.87, η^2^_G_ < 0.001, nor a significant condition*time interaction, *F*(3.88, 186.43) = 0.58, ε = 0.61, *p* = 0.67, η^2^_G_ = 0.002.

#### Heart rate

Regarding heart rate over the experiment’s course, the mixed ANOVA with *time* as within factor, revealed a main effect for *time, F*(2.15, 103.31) = 62.40, ε = 0.72, *p* < 0.001, η^2^_G_ = 0.09, and condition*time interaction*, F*(2.15, 103.31) = 3.08, ε = 0.72, *p* = 0.05, η^2^_G_ = 0.01, but no main effect for *group, F*(1, 48) = 2.58, *p* = 0.12, η^2^_G_ = 0.05. Pairwise comparisons revealed a higher heart rate during the TSST-VR preparation phase than in the control condition, *t*(48) = 2.14, *p* = 0.04, (Fig. 2d).

### Discussion

The aim of our first study was to evaluate the subjective and psychobiological stress responses elicited by the Open TSST-VR. We compared the Open TSST-VR and the VR adaptation of the placebo TSST as an active control condition regarding subjective stress ratings, salivary cortisol, salivary alpha-amylase, and heart rate.

Overall, the Open TSST-VR resulted in significantly higher subjective stress ratings and in an increased heart rate than in the active control condition. Notably, there were more cortisol responders in the stress condition, although we observed a high number of non-responders in both conditions and a wide range in the baseline-to-peak responses in reaction to the TSST-VR. For the average salivary cortisol response, the group difference between the TSST-VR and the active control condition was insignificant, indicating a similar response to the stimulation in both conditions in VR. There were no differences between the conditions regarding alpha-amylase.

These results are consistent with previous studies using the TSST in VR, which also reported pronounced stress responses in heart rate and subjective stress ratings and, to a lesser extent, in salivary cortisol (Liu & Zhang, 2020; Shiban et al., 2016). In addition, these effects concur with the results of recent meta-analyses (Dammen et al., 2022; Helminen et al., 2019) showing that overall, VR adaptations of the TSST elicit a weaker cortisol reaction than the in vivo versions. Therefore, two questions arise. Why do VR versions of the TSST tend to have a smaller effect on HPA axis activation? And why did the stress and control conditions not differ as clearly as expected?

We can answer the first question by stating that the TSST-VR differs somewhat from the traditional face-to-face versions. In our version, however, we modified the VR protocol to resemble as closely as possible the in vivo versions of Kirschbaum et al. (1993) and of Het et al. (2009) for the placebo version. Immersiveness and the feeling of presence are known to be key factors in how virtual environments are perceived (Dammen et al., 2022). Although we used a VR system suitable to implement an immersive effect and have already shown that the sense of presence appears to be quite robust against inconsistencies between the real lab context and VR environment created (Zimmer et al., 2019b), there might have been some aspects in the experimental procedure that might have disturbed the immersion and feeling of presence, e.g., the saliva sample while wearing the HMD. Another factor potentially influencing the sense of presence is that the experimenter was in the same room during the entire experiment. Although the experimenter and participants did not interact during the procedures in VR, it is possible that this presence might have affected the ability to forget “reality” and feel present in the virtual environment.

Related to the second question, namely why the control condition elicited some cortisol reactions resembling those observed in the stress condition, it is possible that some participants might have found the VR environment or a VR lab’s experimental atmosphere to be stress-inducing. Initial indications of this may be slightly increased subjective stress and measurable HR response to the control condition. We cannot rule out that some participants may have found the VR environment so unfamiliar that the mere novelty of this environment triggered a measurable arousal and, in some cases, increased activity of the HPA axis. Future studies could address this problem by familiarizing participants with the VR environment before the actual experiment and allowing sufficient habituation time. Another possible approach to investigate the effects of the VR environment is the implementation of a repeated measures design. In that case, one would expect the first session to always reveal more stress potential.

To conclude, in this first study, the Open TSST-VR resulted in a significant increase in subjective stress ratings and in the heart rate, with a weaker effect on the HPA axis. Besides these initial findings with a novel open version of the TSST-VR, open questions and limitations remain. First of all, only men were tested in the first study, which limits the generalizability of our results. Secondly, with the group design we used, we cannot rule out that the specific effect of stress was masked by general novelty effects of the VR environment.

## Study 2: Replication and gender differences

Due to these considerations and implications, we conducted a second study with an independent sample of participants and adaptations to replicate and extent our initial findings. In study two, we included naturally cycling and oral contraceptive-using women to investigate potential sex effects. We used a balanced within-subject design to increase statistical power and explore potential order effects. Finally, we slightly changed the procedures to preserve participants’ VR immersion by skipping the saliva sample during the TSST and placing the experimenter in another room during the VR situation. In general, we expected these changes to improve the TSST-VR’s overall effectiveness compared to the active control condition with responder rates and overall average reactivity closer to prior reports (e.g. Zimmer et al., 2019a).

### Method

All methods were identical to study 1, unless otherwise stated and specified below.

#### Participants

An independent sample of 48 participants was randomly assigned to either start with the TSST-VR or with the control condition. Two data records (both women) were excluded from the analysis due to extreme cortisol values above 30 nmol/l. Our final sample consisted of 46 participants (men/women: *n* = 24/*n* = 22; age range: 19–30 years, *M* = 23; *SD* = 3.11). This second a priori sample size estimation was again based on effects derived from Zimmer et al. (2019a). For the within-subject design, our calculations resulted in a minimum sample size of *N* = 44 (*f* = 0.30; α = 0.05; 1–β = 0.95; correlation for repeated measures *r* = 0.30; correction for non-sphericity Greenhouse–Geisser ε = 0.44). To compensate for possible dropouts, we aimed for a total sample of *N* = 48.

As in study 1, participants were recruited by on-campus advertisement and additionally distributed in public places. Study 2’s inclusion criteria were identical to study 1’s. In addition, women using oral contraceptives (OC) and naturally cycling women were included if their average regular cycle length was between 21 and 35 days. This study was also approved by the ethics committee at the University of Trier and conducted in line with the Declaration of Helsinki. All participants gave informed written consent and were paid 50€ for their participation.

#### Apparatus, questionnaires, and heart rate monitoring

Study 2 followed the same general procedure and used the same technical equipment as study 1. For more information about the questionnaires used, see the supplementary methods.

#### Saliva sampling and biochemical analysis

Saliva samples were collected at seven time points throughout the experiment (– 30, – 16, + 17, + 25, + 35, + 50, and + 65 min in reference to the beginning of the TSST instruction presented in VR). Biochemical analysis was the same as in study 1.

#### Design and procedure

**Fig. 3:**
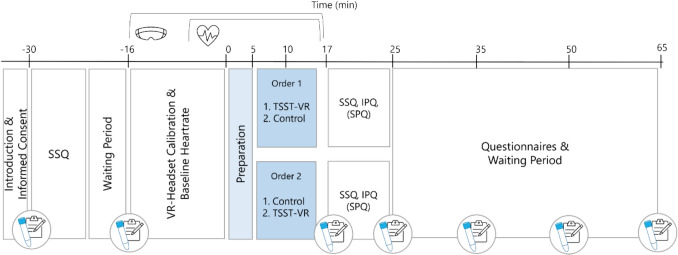
Main procedures of study 2. Timepoint 0 indicates the start of the TSST-VR/Control condition. Heart rate was continuously measured from – 5 to + 16 min. Saliva samples and subjective stress ratings were taken at seven timepoints. *IPQ* Interpersonal Presence Questionnaire, *SPQ* Social Presence Questionnaire, *SSQ* Simulator Sickness Questionnaire

Study 2 followed a single-blind randomized cross-over within-subject design: Half of the study group started with the TSST-VR, half with the control condition. Experimental sessions started at 2:30 p.m. or 4:30 p.m. and were scheduled with 1 week between the two sessions. Participants were asked to refrain from physical exercise, alcohol, caffeine, and painkillers at least 24 h prior to testing, as well as to refrain from consuming anything but water 2 h prior to each appointment. For naturally cycling women, both experimental sessions were scheduled during their luteal phase: the first between 13 and 10 days before the expected start of their next cycle, calculated by counting backward from the expected start of their next cycle (see Schmalenberger et al., 2021). The experimental sessions for OC users were scheduled during the first 2 weeks of OC intake (Fig. 3).

The testing sessions were identical to study 1 except for the following variations. (1) To reduce potential disruptions of the participants’ immersion, no saliva sample was taken right before starting the (placebo) TSST, (2) the experimenter left the main room at the end of baseline measures to control the TSST-VR from an adjacent room, and (3) the participants were first informed about the task’s content during the preparation phase, rather than before the start of the VR segment.

#### Statistical analyses

All statistical analyses were equivalent to study 1, except two more factors were added to the ANOVAs resulting in a 2 (condition) * 2 (order) * 2 (sex) * 7 (time) mixed design with *condition* and *time* as within-subject factors, and *sex* and *order* of the testing sessions as between-subject factors.

### Results

#### Baseline differences simulator sickness, feeling of presence, and experience with VR

There were no differences in age, BMI, psychological symptoms, fear of negative evaluation, social anxiety, chronic stress, and feeling of personal or social presence in the VR environment between participants starting with the TSST-VR and starting with the control condition (all *p* > 0.09). According to Fisher’s exact test, there were no differences between the two subgroups (*p* = 1) and no sex differences (*p* = 0.08) regarding prior VR experience.

For female participants, we checked for possible positive and negative menstrual changes between participants starting with the TSST-VR and starting with the control condition, and between the testing sessions. ANOVA revealed no significant effects (all *p* > 0.6).

#### Subjective reaction

**Fig. 4:**
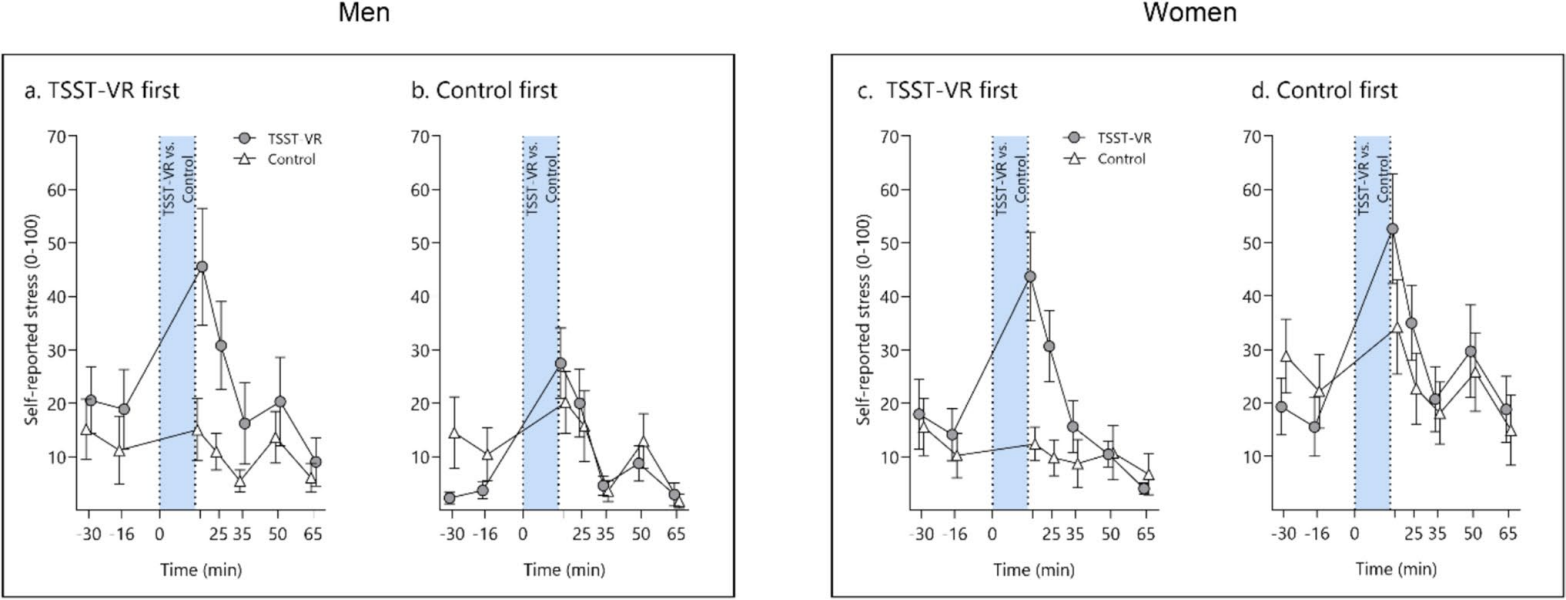
Self-reported stress to the Open TSST VR in men starting with (**a.**) the TSST-VR vs. (**b.**) with the control condition and women starting (**c.**) with the TSST-VR vs. (**d.**) with the control condition

Missing data from two participants in at least one measurement point resulted in exclusion from this analysis. The mixed ANOVA for the VAS item “How stressed do you feel at the moment” showed significant main effects for the *condition, F*(1, 40) = 4.97, *p* = 0.031, η^2^_G_ = 0.02, and *time, F*(3.13, 125.34) = 30.53, ε = 0.52, *p* < 0.001, η^2^_G_ = 0.13, as well as for the two-way interaction between *condition* and *time, F*(3.42, 136.89) = 18.48, ε = 0.57, *p* < 0.001, η^2^_G_ = 0.05, and the three-way interaction of *order*, *time* and *condition, F*(3.42, 136.89) = 2.95, ε = 0.57, *p* = 0.03, η^2^_G_ = 0.01. No other effects were significant (all *p* > 0.05). Pairwise comparisons (collapsed over *sex*) revealed significantly higher subjective stress ratings in the stress than the control condition at the sampling point at + 17, *t*(40) = – 6.29, *p* < 0.0001, and + 25 min, *t*(40) = 4.81, *p* < 0.0001, in participants starting with the TSST-VR. In contrast, participants starting with the control condition showed a significant difference at the first measurement point (– 30 min), *t*(40) = – 2.36, *p* = 0.02, suggesting that they were more stressed on the first testing day. However, this subgroup exhibited higher TSST-VR levels at + 17 min, *t*(40) = – 2.31, *p* = 0.03 (see Fig. 4).

#### Free salivary cortisol

**Fig. 5:**
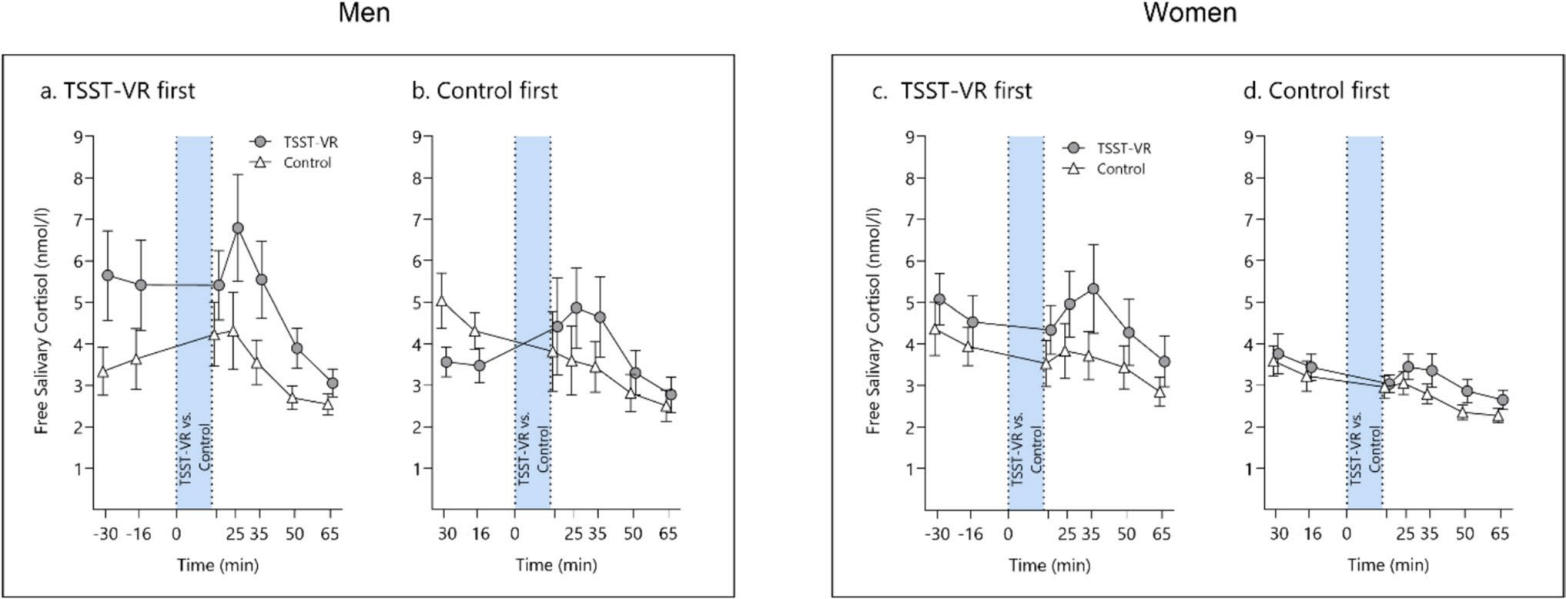
Cortisol response to the Open TSST VR in men starting **(a.**) with the TSST-VR vs. (**b.**) with the control condition and women starting (**c.**) with the TSST-VR vs. (**d.**) with the control condition

The mixed ANOVA revealed a significant mean effect for *time, F*(1.89, 79.52) = 9.08, ε = 0.32, *p* < 0.001, η^2^_G_ = 0.06, and *condition, F*(1, 42) = 13.65, *p* < 0.001, η^2^_G_ = 0.03, and for the interaction order*condition, F(1, 42) = 5.70, *p* = 0.02, η^2^_G_ = 0.01. No other effects were significant (all *p* > 0.09). Pairwise comparison of this interaction revealed that the *condition* effect was only significant in participants who started with the TSST-VR procedure, *t*(42) = – 4.41, *p* = 0.0001. We again applied the 2.5 nmol/l baseline-to-peak criterion to distinguish responders from non-responders in salivary cortisol. Thirteen (28.3%; four women) participants showed this response after the TSST-VR and four (8.7%; one woman) after the placebo procedure. Fisher’s exact test confirmed this difference (*p* = 0.03) (Fig. 5).

#### Salivary alpha-amylase

For salivary alpha-amylase, results showed a significant main effects for *time, F*(3.58, 150.48) = 8.98, ε = 0.60, *p* < 0.001, η^2^_G_ = 0.03, and *sex, F*(1,42) = 8.30, *p* = 0.01, η^2^_G_ = 0.14. No other effects were significant (all *p* > 0.1).

#### Heart rate

**Fig. 6:**
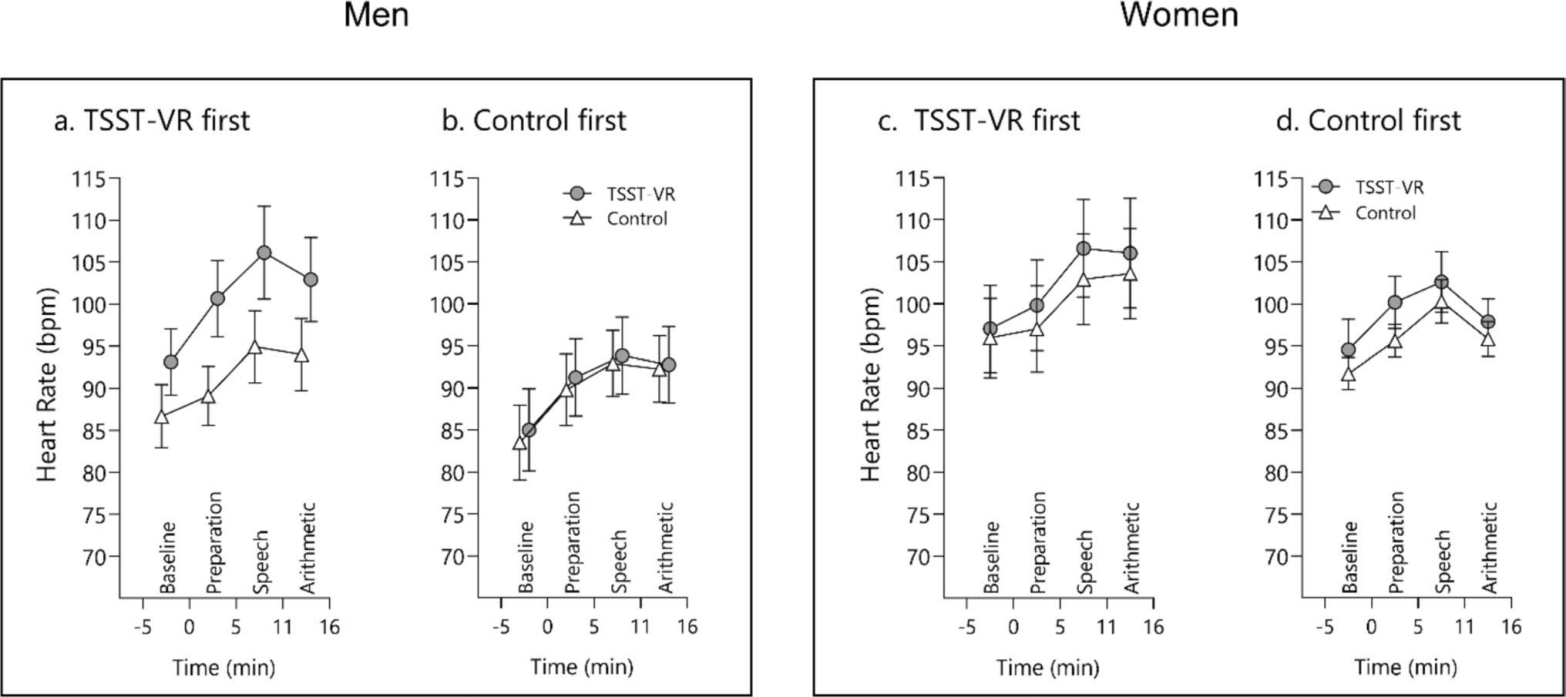
Heart rate reaction to the Open TSST VR in men starting (**a.**) with the TSST-VR vs. (**b.**) with the control condition and women starting (**c.**) with the TSST-VR vs. (**d.**) with the control condition

Data from two participants were excluded due to missing data. The mixed ANOVA for HR resulted in a significant mean effect for *time, F*(1.86, 74.25) = 50.68, ε = 0.62, *p* < 0.001, ε = 0.63, η^2^_G_ = 0.06, and the interactions sex*condition, F(1, 40) = 4.17, *p* = 0.05, η^2^_G_ = 0.01, and order*condition, F(1, 40) = 6.52, *p* = 0.02, η^2^_G_ = 0.02. No other effects were significant (all *p* > 0.06). Pairwise comparisons showed that only for men, *t*(40) = – 2.84, *p* > 0.01, and for participants starting with the TSST-VR, *t*(40) = – 3.14, *p* > 0.01, there was a significant effect of the condition, with higher HR during the TSST-VR than the control condition (see Fig. 6).

### Discussion

With the second study, we aimed to replicate and extent our first study’s findings. An independent sample of men and women was tested with the Open TSST-VR and the control condition in a cross-over within-subject study. This design enabled us to explore order effects and sex differences.

For participants starting with the stress condition, the Open TSST-VR resulted in a significant rise in not only subjective stress ratings and in heart rate but also salivary cortisol in comparison to the placebo version. However, when the placebo TSST-VR took place first, the difference between the stress and control conditions was only marginal or non-existent. In other words, the cortisol stress response to the TSST-VR seems to be stronger when participants were exposed to the stress condition first. In turn, the observed pattern of subjective stress responses suggests that the control condition also elicited stress in at least some participants. One explanation for this order effect is a potential interaction of the cortisol response’s well-known habituation to repeated TSST exposure both in vivo (Boesch et al., 2014; Kexel et al., 2021) and in VR (Kothgassner et al., 2021), technostress due to the unfamiliarity of the VR technology and potential ergonomic issues with the HMD (Khan et al., 2024), the unfamiliarity of experimental procedures itself, and the intended effects of the stress and control conditions. When exposed to the stress condition first, the stress-inducing elements of the TSST and technostress might have worked together to produce the observed stress response, while during the second VR session with the placebo TSST, habituation to both the experimental setup and the VR technology and the non-stressful tasks might have resulted in the observed weaker overall response. Comparatively speaking, the significant stress reaction to the control condition during the first VR session would be a product of technostress and the novelty of the experiment itself, while the reduced but still significant reaction to the stress condition during the second VR session would be based in the interaction of the TSST’s stress-inducing elements and habituation to the VR technology and experimental setup. That would mean that the OpenTSST-VR itself works as intended, but needs an adjusted procedure that reduces technostress, e.g., by allowing participants to interact with the VR technology before applying any TSST condition.

Besides this order effect, there were only subtle differences between men and women. There were only marginal differences in their subjective response and cortisol response, which were statistically not significant. We detected no effects on alpha-amylase. Similarly, we identified no significant sex differences regarding personal and social presence during the VR task or general VR experience.

However, men showed a significantly stronger increase in their HR response to the TSST-VR when it was presented in the first session, while the women did not reveal such a significant difference. Moreover, we detected no sex effect at all. This result contradicts previous findings (Liu & Zhang, 2020), who observed a general sex effect with a significantly weaker HR response after the VR stressor in men compared to OC-free and menstrual phase-balanced women. This is in line with descriptive results from Kirschbaum et al. (1999), where men showed the lowest HR stress reactivity compared to women in the follicular phase, OC users and women in the luteal phase. In our study, we balanced OC users and NC women, and standardized assessment times (with OC users being invited in the first 2 weeks of OC intake and NC women being in the luteal phase), but were unable to test for effects of OC usage and menstrual phase standardization due to our generally smaller sample. Thus, our results need further confirmatory testing in bigger samples that enable tests for effects of OC usage vs. natural cycling, and follicular vs. luteal phases. The Open TSST-VR therefore appears to be less susceptible to sex differences, although we must bear in mind that these results are only preliminary considering our relatively small sample and the fact that our entire sample was examined in two different sequences. Another limitation is our heterogeneous female sample in their hormonal contraception and cycle phase. Both factors could have an influence, but this might be only detectable in larger samples and with systematic variation (Gervasio et al., 2022).

## General discussion

The Open TSST-VR is a freely available VR version of the classic in vivo TSST that is easily adaptable (e.g., spoken language) and thus suitable for large-scale collaborative studies. In two independent studies, we evaluated the Open TSST-VR by comparing the TSST-VR to an active control condition (the placebo TSST-VR). Our results from both studies provide consistent evidence for significant subjective psychological stress reactions to the stressor in all participants regardless of sex. In addition, the Open TSST-VR taken by male participants resulted in significant changes in heart rates, indicating autonomic nervous system activation. As for the heart rate in female participants and alpha-amylase in general, however, we found no evidence for a significant reaction to the Open TSST-VR. Regarding HPA axis activation as measured by free salivary cortisol, both studies revealed only modest average reactivity, with an overall cortisol-responder rate to the Open TSST-VR of approximately 30%. However, both studies provided evidence that the control condition also elicited subjective stress and cortisol reactions to a lesser extent.

Compared to other evaluations of the VR versions of the TSST (e.g., Zimmer et al., 2019a), the physiological stress effects in the studies described above were less pronounced. This is consistent with an earlier meta-analysis that concluded that the virtual stressor “elicited a varied magnitude of physiological stress reactivity” and had an overall medium effect size (Dammen et al., 2022). There are at least three possible explanations: (1) the Open TSST-VR by itself could simply lack the intensity needed to elicit a significant endocrine stress reaction. This seems plausible since other VR-TSST stressors also report significantly reduced cortisol reactivity (e.g., Kelly et al., 2007). Instead of simply attempting to up the intensity by adjusting the jury interaction or task parameters alone, however, future research should try to objectively measure stressor intensity, e.g., by assessing changes in skin conductance and pupil dilation (Armario et al., 2020). Reliable pupillometry has been evident in the use of an off-the-shelf VR headset (Eckert et al., 2021), and could be integrated in natural, interactive eye-tracking (e.g., Vehlen et al., 2021), enabling direct comparisons between VR and in vivo TSSTs; (2) there are differences in how the Open TSST-VR is perceived compared to the in vivo TSST that reduce the endocrine stress reaction. Goodman et al. (2017) reported that negative jury feedback during in vivo TSSTs results in on average significantly weaker salivary cortisol reactivity than neutral feedback. Due to the limited visual quality of VR rendering, it is possible that participants read VR jury faces in the Open TSST-VR as negative and thus demonstrate less stress reactivity. This could be addressed by assessing the emotional valence participants perceive, as well as enhancing visual face clarity by improving rendering and lighting adjustments in VR; and (3) there are factors other than the interaction with the virtual panel that drive the stress reaction. In the Open TSST-VR, the experimenter is the only other human with whom the participants interact. Use of another socially evaluative stressor, the SECPT, revealed that the experimenter’s behavior and demeanor can have a major impact on the cortisol reaction (Schwabe & Schächinger, 2018). The salience of the experimenter’s behaviors could be similar in the Open TSST-VR and thus have a similar effect on the participants’ physiological and psychological stress responses: without the presence and “real-life threat” of a living jury, neutrally interacting experimenters themselves could invoke the feeling of control loss and evaluative threat produced by the human jury in the in vivo TSST. This could result in a stress response by experiencing shame (Gruenewald et al., 2004). It would also help explain why participants showed a significant stress response in the control condition: experimenters were trained to always interact neutrally and professionally, and their proximity to the experiment (staying in the room while the TSST vs. leaving the room) was kept consistent, thus being a constant in the experimental conditions. Thus, details regarding the experimenter and their interaction in a VR study should be systematically varied in the future to allow for effect testing. In addition, it also is possible that the reduced (cortisol) stress response relative to the control condition is at least partly due to the unspecific reaction to the VR itself as a novel, unfamiliar environment that could result in technostress. This interpretation concurs with the already elevated baseline salivary cortisol levels in some participants, and the fact that there were also some responders in the control condition and the elevated subjective stress in both conditions. This would call for longer adaptation periods in the VR setting, for example by adding a non-stressful VR task before the TSST, to allow for participants’ familiarization with the setting and normalization of baseline salivary cortisol levels. Future studies could also include a passive (low-level) resting control condition to control for the effects of the VR environment or implement the friendly TSST (Wiemers et al., 2013; Kurz et al., 2024) as a non-threatening, but structurally and demand-wise similar control condition.

## Conclusion

Overall, the present study provides initial evidence for the general suitability of the Open TSST-VR to induce moderate levels of psychosocial stress in men and women. As an open, freely usable, and readily adaptable VR version, it has the potential to promote collaborative research and large-scale investigations into the antecedents, moderators, and consequences of acute stress in health and disease and in different age groups. Future research should investigate potentially interfering factors to improve the Open TSST-VRs efficacy as a psychophysiological stressor.

## Data Availability

The materials related to this paper are available at OSF: https://osf.io/cmw2g/.

## Code availability

The Users’ Guide, executable files of the Open TSST-VR, and analysis code related to this paper are available at OSF: https://osf.io/cmw2g/.
